# Mebendazole Impedes the Proliferation and Migration of Pancreatic Cancer Cells through SK1 Inhibition Dependent Pathway

**DOI:** 10.3390/molecules27238127

**Published:** 2022-11-22

**Authors:** Khem Raj Limbu, Rashmi Bhandari Chhetri, Yoon Sin Oh, Dong Jae Baek, Eun-Young Park

**Affiliations:** 1 College of Pharmacy, Mokpo National University, Mokpo 58554, Republic of Korea; 2 Department of Food and Nutrition, Eulji University, Seongnam 13135, Republic of Korea

**Keywords:** mebendazole, pancreatic ductal adenocarcinoma, sphingosine kinase, sphingosine 1-phosphate

## Abstract

Pancreatic ductal adenocarcinoma (PDAC) has one of the highest mortality rates and requires the development of highly efficacious medications that can improve the efficiency of existing treatment methods. In particular, in PDAC, resistance to conventional chemotherapy reduces the effectiveness of anticancer drugs, decreasing the therapeutic efficiency. Sphingosine 1-phosphate (S1P), produced by sphingosine kinase (SK), plays a vital role in cancer growth, metastasis, chemotherapy, and drug resistance. Focusing on the structural characteristics of mebendazole (MBZ), we studied whether MBZ would affect metastasis, invasion, and drug resistance in cancer by lowering S1P production through inhibition of SK activity. MBZ selectively inhibited SK1 more than SK2 and regulated the levels of sphingolipids. MBZ inhibited the proliferation and migration of cancer cells in other PDAC cell lines. To determine whether the effect of MBZ on cancer cell growth and migration is S1P-mediated, S1P was treated, and the growth and migration of cancer cells were observed. It was found that MBZ inhibited S1P-induced cancer cell growth, and MBZ showed a growth inhibitory effect by regulating the JAK2/STAT3/Bcl-2 pathway. The phosphorylation of focal adhesion kinase (FAK), a transcription factor that regulates migration, was inhibited by MBZ, so it was found that the effect of MBZ regulates the migration of cancer cells through the S1P/FAK/vimentin pathway. In conclusion, our study suggests that the anthelmintic MBZ can be used as a potential therapeutic agent for treating PDAC and for structural synthesis studies of its analogs.

## 1. Introduction

Pancreatic cancer is one of the deadliest and most aggressive malignancies, with 495,773 new cases reported worldwide in 2020 and 466,003 deaths due to pancreatic cancer. In particular, pancreatic ductal adenocarcinoma (PDAC) has a terrible prognosis with a five-year survival rate of less than 10% [1,2,3]. The asymptomatic nature of PDAC in the early and late stages of diagnosis, the lack of a precise diagnosis, and drug resistance to chemotherapy, increase the mortality rate of PDAC [4,5,6]. Pancreatic cancer can be treated with chemotherapy, radiation therapy, and surgery, and in the case of late-stage PDAC, chemotherapy remains the only option for treatment [7]. Gemcitabine-based combinations with other medications are mainly used for PDAC therapy. However, as a treatment for PDAC, the efficacy may decrease due to problems such as tolerance, genetic mutation, and tumor microenvironment, which plays a critical role in drug resistance [8,9,10], so there is a need to develop more effective drugs with a different mechanism of action [11]. However, long-term studies may require new medications to control this devastating disease. Repurposing existing drugs approved for other diseases may help overcome the urgency of discovering novel anticancer drugs [12,13,14].

The Food and Drug Administration (FDA) has approved the drug mebendazole (MBZ), which was first developed to manage helminthic parasites. In a recent study, it has reportedly been shown to have an anticancer effect on various cancer types in vitro and in vivo [15,16,17]. MBZ’s primary mechanism of action is the depolymerization of tubulin, although other investigations have found that its derivative, Fenbendazole, destabilizes tubulin polymerization by stabilizing P53 and causing apoptosis [18,19]. MBZ has been shown to inhibit multiple signaling pathways, including MAPK/STAT3, JNK, ELK/SFR, MYC/MAX, and NF-κB, leading to apoptosis, autophagy, and DNA damage in various types of cancer, including pancreatic cancer. [20,21]. The metastasis of PDAC can be decreased by MBZ alone or in combination [17]. In vivo studies have shown that MBZ has tumor-suppressive effects in early and late pancreatic cancer models and reduces metastasis to other organs [15]. Based on these studies, MBZ alone or in combination with other anticancer drugs can be applied as a therapeutic agent for PDAC. A mechanism-based study for this may be essential.

Sphingosine kinases phosphorylate sphingosine to produce sphingosine 1-phosphate (S1P), and in mammalian cells, sphingosine kinases have two isomers, sphingosine kinases 1 and 2 (SK1 and SK2), respectively [22,23]. Sphingosine 1-phosphate (S1P) plays a crucial role in cancer cell proliferation, migration, angiogenesis, and invasion [24,25] through G-protein coupled S1P specific receptors 1–5 [26]. On the other hand, sphingolipids, such as ceramide and sphingosine, inhibit cell growth and increase apoptosis [27,28,29,30]. In addition to these, several exogenous stimuli, such as transforming growth factor-beta (TGF-β), vascular endothelial growth factors (VEGF), and epidermal growth factor (EGF), work through the receptor tyrosine kinase to stimulate SK1 and carry out the extracellular-signal-regulated kinase (ERK) signaling pathway [31]. In addition, several signaling pathways, including MAPK/JNK and JAK2/STAT3, for SK1 in cancer have been described [32]. Several studies have demonstrated that SK inhibition prevents the metastasis of several cancers, including pancreatic carcinoma [33,34,35], where the overexpression of SK1 is associated with chemotherapeutic drug resistance, and inhibiting SK1 could be a potential strategy to treat PDAC patients who have developed drug resistance [36]. In a related study, SK1 knockdown altered chemotherapy resistance in lung cancer and osteosarcoma [37,38]. Therefore, the inhibition of SK can be presented as an action point in overcoming the therapeutic limitations caused by drug resistance of existing pancreatic cancer therapeutics. SKI-349 was announced as a chemical with a dual target that inhibits SK activity and microtubule polymerization [39]. SKI-349 and MBZ have structurally similar features. Both compounds have a benzoyl backbone, SKI-349 has a characteristic thiazole structure, and MBZ contains a similar imidazole structure. This study aimed to examine whether the cytotoxic effect of MBZ on PDAC cells was dependent on SK activity, focusing on the fact that the structure of MBZ has structurally similar characteristics to that of the SK inhibitor SKI-349.

## 2. Results

### 2.1. MBZ Inhibits SK Activity and Thereby Reduces the Production of S1P in PDAC Cells

**Figure 1:**
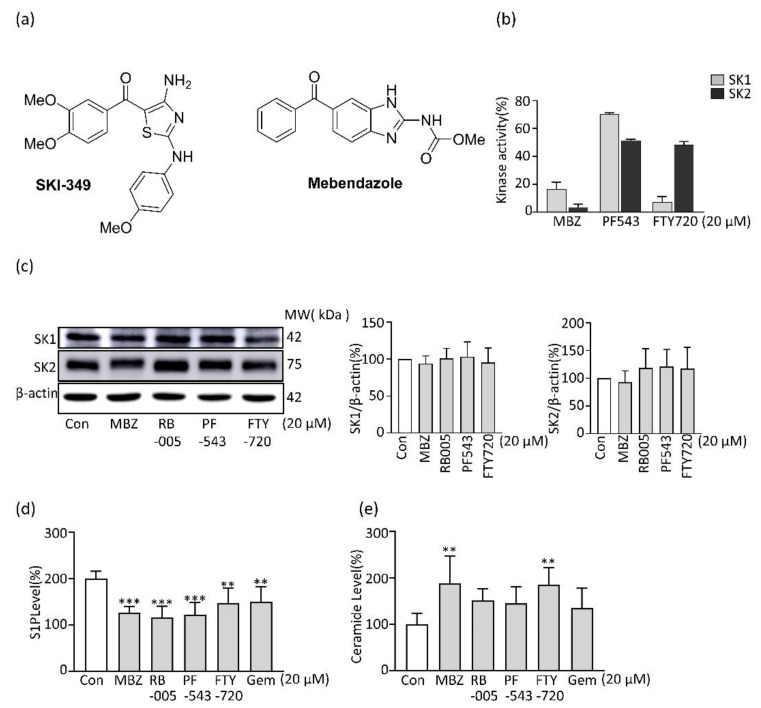
Evaluation of SK activity and sphingolipids of MBZ: (**a**) structure of SKI-349 and MBZ; (**b**) SK1 and SK2 activity of MBZ, PF543 and FTY720 at 20 µM concentrations; (**c**) SK1 and SK2 expression levels of MBZ, RB005, PF543 and FTY720 (20 µM), protein level was normalized with β-actin; results were quantified in a ratio of SK1/β-actin and SK2/β-actin using ImageJ software; (**d**,**e**) measurement of sphingosine-1-phosphate and ceramide levels in MIA PaCa-2 cells after treatment with 20 µM compounds for 24 h. Each experiment has performed a minimum of thrice and the data were illustrated as mean ± S.D. ** *p* < 0.01 and *** *p* < 0.001 compared with the control group.

Considering that MBZ has a structural similarity to the SK inhibitor SKI-349, the effect of MBZ on SK activity was analyzed. As a result of testing SK1 and SK2 inhibitory efficacy with PF543, which is known as the most potent SK1 inhibitor, and FTY720, which is known to have SK1 inhibitory efficacy, as a control group, MBZ showed higher SK1 inhibitory activity than FTY720. It was found that the activity of MBZ was selective for SK1 rather than SK2 (Figure 1b). Furthermore, we checked the expression of SK1 and 2 at the protein level to find out whether MBZ suppressed the activity of SK through suppression of SK protein expression. The protein levels of SK1 and SK2 were not changed by MBZ treatment (Figure 1c). Since SK changes sphingosine to S1P, which is known to promote the proliferation of cancer cells, we measured the levels of S1P after MBZ treatment. According to our findings, MBZ treatment lowers S1P levels through the SK inhibitory effect while increasing pro-apoptotic ceramide levels (Figure 1d,e).

### 2.2. MBZ Inhibits the Viability, Proliferation, and Migration of Pancreatic Cancer Cell Lines

**Figure 2:**
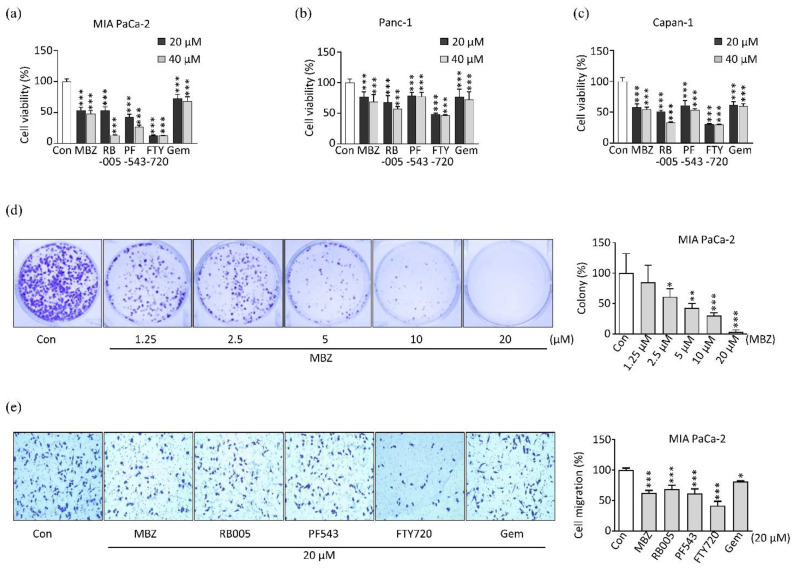
Mebendazole inhibits the viability, proliferation and migration of PDAC cell lines: (**a**–**c**) cytotoxic effect exerted by MBZ, RB005, PF543, FTY720 and gemcitabine treated with 20 and 40 µM each on PDAC cell lines: MIA PaCa-2, Panc-1 and Capan-1, respectively; (**d**) colony formation in MIA PaCa-2 cell line after 24 h treatment with different concentrations (1.25 µM, 2.5 µM, 5 µM, 10 µM and 20 µM) of MBZ; (**e**) transwell migration of MIA PaCa-2 cell after 24 h treatment with 20 µM concentration of MBZ, RB005, PF543, FTY720 and gemcitabine. All experiments were carried out three times and data were illustrated on mean ± S.D, * *p* < 0.05, ** *p* < 0.01 and *** *p* < 0.001.

We tested whether MBZ inhibits cell viability and proliferation in pancreatic cancer. For this, we performed 3-[4,5-dimethylthiazol-2-yl]-2,5-diphenyltetrazolium bromide (MTT) assay for viability with 20 μM and 40 μM chemical concentrations in different pancreatic cancer cell lines; MIA PaCa-2, Panc-1, and Capan-1. We found that MBZ significantly represses the viability of pancreatic cancer cell lines (Figure 2a–c). MBZ exhibited a high cytotoxic effect in MIA PaCa-2, and Capan-1 cell lines compared to Panc-1 cell lines and had higher cytotoxic activity compared to that of gemcitabine and lower than that of other SK1 inhibitors like RB005 [40] and FTY720 in the same concentration (*p* > 0.05). However, in Panc-1 and Capan-1 cell lines, MBZ had a similar effect to the potent SK1 inhibitor PF543 [41]. Then, we further investigated the effect of MBZ on pancreatic cell proliferation in the MIA PaCa-2 cell line using a colony-forming assay. MBZ significantly inhibits cell proliferation in a dose-dependent manner (Figure 2d). The 2.5 μM concentration significantly inhibited the proliferation of pancreatic cancer cells with about 61% surviving factor, followed by 5 μM, 10 μM and 20 µM concentrations of MBZ treatment, with a decline of the survival fraction of around 43%, 30.4%, and 3.5%, respectively (*p* > 0.05). Collectively, these outcomes illustrate that MBZ can impede pancreatic cancer cell proliferation and viability.

After observing the antiproliferative effect of MBZ treatment, we tested MBZ for its effect on cell migration in the MIA PaCa-2. At a concentration of 20 μM, MBZ significantly reduced cancer cell migration, similar to the gemcitabine and the SK1 inhibitor RB005 (Figure 2e). Diverse studies found similar results in oral, gastric, colon, ovarian, and head and neck cancer [20,42,43,44]. These outcomes suggested that MBZ suppressed the migration and proliferation of pancreatic cancer cells.

### 2.3. MBZ Induces Apoptosis in Pancreatic Cancer Cells through the Intrinsic Mitochondrial Pathway

**Figure 3:**
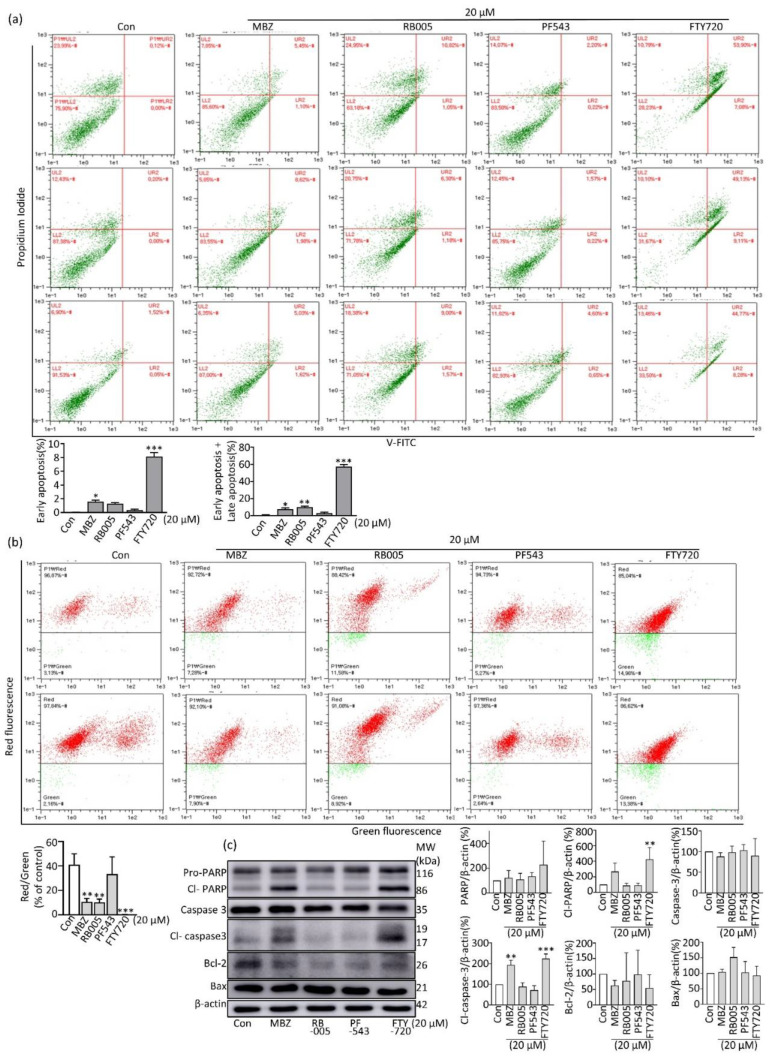
Mebendazole promote apoptosis in pancreatic cancer cell through intrinsic mitochondrial pathway: (**a**) MIA Paca-2 cell line was treated with desired concentrations using MBZ, RB005, PF543 and FTY720 treatment for 24 h and apoptosis was analyzed by Annexin V-FITC; (**b**) JC-10 assay was performed to determine mitochondrial membrane potential (MMP) after treatment at a concentration of 20 µM; (**c**) analysis of apoptosis marker using western blotting and ImageJ software was used to quantify western blotting results and data were normalized with β-actin. Each experiment was repeated three times independently. Data were expressed on mean ± S.D, * *p* < 0.05, ** *p* < 0.01 and *** *p* < 0.001.

Different anticancer medicines cause cell death mechanisms in distinct malignancies. Among them, apoptosis is one of cancer’s most critical strategies to program cell death [43]. We therefore looked at whether MBZ could cause apoptosis in pancreatic cancer cells. A result of flow cytometry (FACS) analysis revealed that apoptotic cells were increased by MBZ when compared to the control group (Figure 3a) (* *p* < 0.05 to *** *p* < 0.01). In addition, we performed JC-10 mitochondrial membrane potential analysis in MBZ-treated MIA PaCa-2 PC cell line at a concentration of 20 μM to determine whether the mitochondrial pathway is involved in the apoptosis pathway after MBZ treatment. As a result, the red/green ratio was significantly reduced (Figure 3b), indicating the presence of an intrinsic mitochondrial pathway with an elevation of mitochondrial membrane potential (MMP). This was confirmed by western blotting, where MBZ treatment significantly increased pro-apoptotic proteins, cleaved PARP, cleaved caspase-3, and decreased anti-apoptotic protein Bcl-2 (Figure 3c). Collectively, these findings suggest that MBZ promotes intrinsic mitochondrial apoptosis in PDAC cells.

### 2.4. MBZ Partially Reverses Apoptosis Inhibited by S1P via the JAK2/STAT3/Bcl-2 Pathway

**Figure 4:**
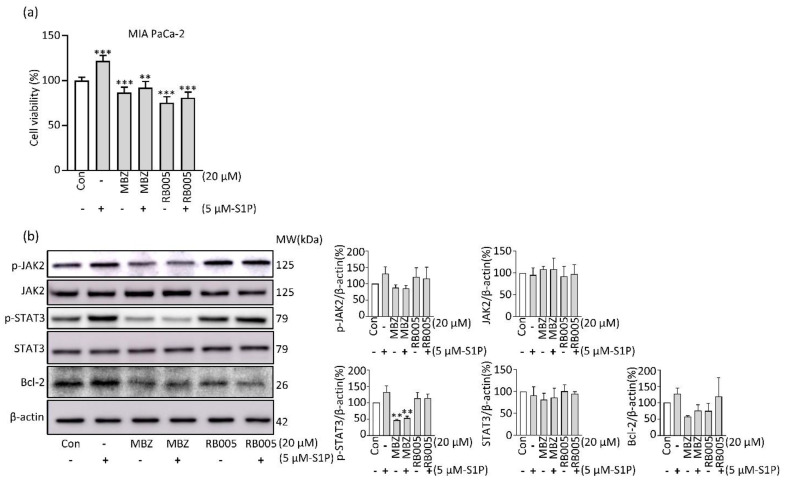
S1P treatment partially reverse the effect of MBZ in PDAC: (**a**) percentage of cell viability in MIA PaCa-2 cells was calculated using 20 µM MBZ, RB005 and combination with 5 µM S1P; (**b**) Western blotting was performed to detect markers related to the JAK2/STAT3/Bcl-2 signaling pathway in PDACs treated with or without 5 μM S1P. Results were quantified by using ImageJ and each experiment was performed independently three times. The data were depicted on mean ± S.D, ** *p* < 0.01 and *** *p* < 0.001.

We found that MBZ had an inhibitory effect on SK activity, thereby reducing S1P levels. In order to investigate whether the cancer cell growth caused by MBZ is S1P-mediated, we observed the effect of MBZ on cell toxicity after S1P treatment. When pancreatic cancer cells were treated with S1P at a concentration of 5 µM, the proliferation of cancer cells was promoted. It was observed that the induced proliferation was inhibited when treated with MBZ (Figure 4a). In addition, we observed changes in the activity of transcription factors to investigate the mechanism involved in the inhibition of MBZ in the cancer cell proliferation effect induced by S1P. As a result of examining the phosphorylation of JAK2 and STAT3, which are involved in the growth and death of cancer cells, phosphorylation was increased in the 5 μM S1P treatment group. It was found that the increased phosphorylation was decreased by MBZ treatment (Figure 4b). Since the expression of the anti-apoptotic marker Bcl-2 is regulated by JAK2 and STAT3, we observed changes in the expression of Bcl-2 by S1P and MBZ. It was observed that the expression of Bcl-2 increased upon S1P treatment and was suppressed in combination with MBZ. These findings suggest that MBZ is involved in the JAK2/STAT3/Bcl-2 pathway activated by S1P to inhibit apoptosis of PDAC.

### 2.5. MBZ Prevents PDAC Cell Migration via Suppression of the S1P/FAK Signaling Pathway

**Figure 5:**
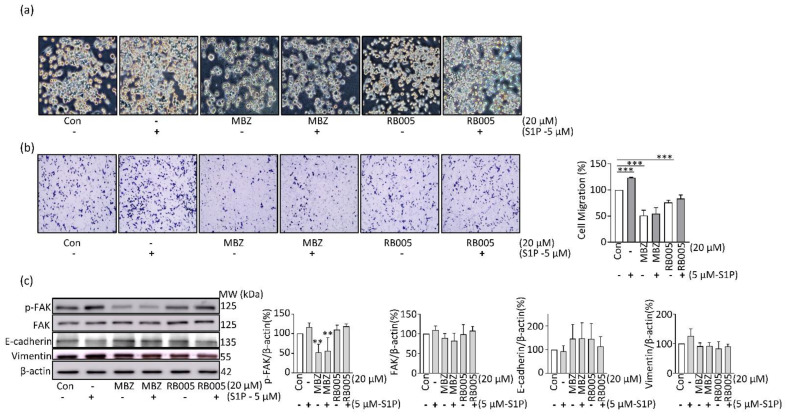
MBZ suppresses migration of PDAC by inhibition of S1P/FAK signaling pathway: (**a**) the MIA PaCa-2 cell treated with and without the addition of 5 µM of S1P plus 20 µM of each MBZ and RB005 is shown in a Phase contrast picture (10×); (**b**) MIA PaCa-2 cell was treated with 20 µM of MBZ and RB005 each separately and along with the combination of 5 µM of S1P for transwell migration assay; (**c**) evaluation of cell migration related markers by Western blotting after the protein extraction after treatment without or with 5 µM of S1P in MBZ and RB005 treated cells. The immunoblot was repeated thrice and data was quantified by the use of ImageJ software. The data were interpreted on mean ± S.D, ** *p* < 0.01 and *** *p* < 0.001.

MBZ was observed to inhibit PDCA cell migration, as shown in Figure 2c, and S1P was introduced to determine whether the MBZ inhibitory mechanism was SK/S1P-dependent. As a result of the transwell migration assay, MIA PaCa-2 cell migration was increased as a result of treatment with 5 μM S1P, and the treatment of MBZ inhibited S1P-induced migration (Figure 5a,b). Since FAK can regulate the expression of genes involved in the migration of cancer cells, we observed changes in the level of FAK phosphorylation. Phosphorylation of FAK was increased by S1P treatment but decreased upon treatment with MBZ alone and S1P and MBZ combination. Upon observing the expression of E-cadherin, a downstream target regulated by FAK, it was observed that the expression of E-cadherin decreased by S1P was increased by MBZ. In addition, due to the expression of vimentin, a gene involved in migration, and a sub-protein of FAK, it was observed that the expression increased by S1P was decreased by MBZ treatment (Figure 5c). These findings suggest that MBZ inhibits PDAC cell migration by inhibiting the S1P/FAK/Vimentin.

## 3. Discussion

The effectiveness of anticancer therapies is dwindling due to the discovery of several new mechanisms involved in cancer cell progression, resistance to chemotherapy, and metastasis. In addition, developing new targeted compounds specific to cancer is costly due to the long process of drug development, which includes target identification, validation, hit development, lead optimization, preclinical studies, and different clinical trials. It takes a long time for them to go through numerous trials for FDA approval and be available in the market [45]. Drug repurposing has already been approved for some diseases and is gaining popularity due to its minimal side effects and low economic burden in the field of drug development [46]. Several members of the benzimidazole anthelminthic drugs group, like albendazole, fenbendazole, and MBZ, have been repurposed as anticancer drugs [47]. Among them, MBZ, which is an FDA-approved anthelminthic drug, is being tested in different cancers like pancreatic cancer [15], ovarian cancer [21], and thyroid cancer [48]. MBZ is an ideal repurposed medication for anticancer treatment because of its proven pharmacokinetics, efficacy, and optimum toxicity profile [49].

S1P produced from SK1 and SK2 plays several roles, such as cell proliferation, angiogenesis, metastasis, and chemical resistance in pancreatic cancer [48]. The efficacy of MBZ in cancer is known by its anti-tubulin polymerization mechanism. However, the results of studies on other action points are insufficient. In addition, it can have different anticancer mechanisms depending on the structural characteristics of MBZ. SK overexpression has been observed in pancreatic cancer patients, and S1P, which is excessively produced by SK, promotes the cancer cell growth, migration, and the drug resistance of pancreatic cancer patients. SK inhibition can be a potential strategy for overcoming pancreatic cancer, and the MBZ and SKI-349, an SK inhibitor, have structural similarities, so the effect of MBZ on SK activity needs to be conducted [50]. MBZ has an inhibitory effect on SK1 activity, which inhibits the production of S1P, suggesting that it can exhibit SK-dependent anticancer activity. Although several attempts have been made to use SK inhibitors as anticancer agents, such as PF543, a potent inhibitor of SK1, it does not have a sufficient anticancer activity to be used for cancer treatment by increasing the level of sphingosine in vivo. In the case of Opaganib (ABC294640), although the IC_50_ for SK2 inhibition is 60 µM, it has excellent anticancer activity and is being tested in patients with pancreatic cancer, liver cancer, and cholangiocarcinoma [51,52,53]. Therefore, the inhibition of SK1 activity by MBZ suggests a mechanism for the anticancer activity that has not been fully explained and may also provide structural evidence for synthesizing SK inhibitors. [50].

Focal adhesion kinase is a tyrosine kinase responsible for the degradation of p53, a tumor suppressor protein [54]. S1P produced by SK1 regulates the phosphorylation of FAK through binding to the G-protein coupled receptor S1PR2, which regulates the expression of genes that control invasion, angiogenesis, and migration in cancer cells [55]. In addition, S1P/CXCL13 regulates VEGF [55], which is involved in FAK phosphorylation, leading to cell migration and invasion in cancer. In our study, mebendazole inhibits the production of S1P, which inhibits FAK phosphorylation, thereby suppressing the expression of vimentin, which is involved in cancer cell migration. Research results suggest that the inhibition of FAK activation by gemcitabine in PDAC correlates with the inhibition of cancer cell metastasis and improved survival [56]. Therefore, the inhibition of S1P production by MBZ and the resulting regulation of FAK can be suggested as essential targets for suppressing pancreatic cancer metastasis. Since the S1P receptor is involved in this regulation, more studies are required on the correlation between the inhibition of S1P by MBZ and the G-protein-coupled receptor involved.

The JAK2/STAT3 pathway is responsible for differentiation, cell growth, immune function, and activation, forming solid tumors in different cancers. S1P binds to the S1P receptor and activates the JAK/STAT signaling system, which inhibits the death of cancer cells by regulating the expression of apoptosis-related genes. The activation of JAK2 and the resulting dimerization of P-STAT3 regulate gene expressions, such as Bcl2, cyclinD, survivin, and XIAP, which causes cancer cell proliferation and resistance to anticancer drugs [57,58]. The higher expression of JAK2 in PDAC patients was found to be correlated with increased tumor size and lower overall survival [59]. It is also responsible for tumor initiation and drug resistance with the enhancement of clonogenic potential, as reported in the study by Park et al. [60]. It is reported that the inhibition of JAK2/STAT3 phosphorylation enhanced the apoptosis mechanism in PDACs, suggesting that it may be a critical action point in pancreatic cancer [59]. As a result of our experiments, MBZ blocked S1P-induced phosphorylation of JAK2 and STAT3 pathways in pancreatic cancer cells, suggesting that this action may have an anticancer mechanism by regulating the expression of genes involved in the apoptosis of cancer cells. 

MBZ induce apoptosis and inhibit metastasis following the S1P/JAK2/STAT3 and S1P/FAK signaling pathways in PDAC. MBZ exhibits a potent anticancer property with a low toxicity profile. Further study is required to investigate MBZ’s cancer-preventive role in combination with chemotherapeutic compounds to prevent the progression and metastasis of cancer and use it as a potential therapeutic drug for pancreatic cancer treatment.

## 4. Conclusions

Numerous anthelmintic agents of the benzimidazole class have been found to have antitumor effects with distinct cancer cell-specific selectivity. In this study, the novel cancer inhibitory effect of MBZ in PDAC cell lines was investigated. MBZ significantly inhibited PDAC cell line viability, proliferation, and migration. MBZ induces apoptosis by inhibiting the S1P/JAK2/STAT3 signaling pathway in PDAC. In addition, MBZ decreased PDAC migration through the inhibition of the S1P/FAK/Vimentin signaling pathway. As there are few treatment options for late-stage PDPC, further studies using MBZ in combination with chemotherapy are needed to develop an effective treatment approach for PDAC.

## 5. Materials and Methods

### 5.1. Reagents, Antibodies and Chemicals

Dulbecco’s modified Eagle’s mediums (DMEM) were received from Welgene Inc. (Namcheon, Gyeonsangbuk-do, South Korea). Fetal bovine serum (FBS), penicillin-streptomycin (PS) and trypsin 0.25% were received from GE Healthcare Life Sciences HyClone Laboratories (Pittsburgh, PA, USA). EZ-CYTOX was purchased from DoGenBio Co., Ltd. (Seoul, South Korea). The apoptosis detection kit (ApoScan^TM^ annexin V-FITC) was received from Biobud (Cat. No.: LS-02-100, Gyeonggi-do, South Korea). SK1 assay kit was obtained from Echelon Corporation (Santa Clara, CA, USA). PARP, Caspase-3, Bcl-2, Bax, FAK, p-FAK (Tyr397), JAK2, p-JAK2 (Tyr1008), STAT3, p-STAT3 (Tyr705), SK2 and SK1 antibodies were bought from Cell Signaling Technology (Danvers, MA, USA). Similarly, E-cadherin antibody and Anti-vimentin antibody were parched from R&D system (R&D systems, Inc. Minneapolis, USA) and Abcam (Cambridge, United Kingdom) respectively. Anti-β-actin and anti-HRP conjugated secondary antibodies like anti-rabbit, anti-goat and anti-mouse were obtained from Santa Cruz Biotechnology (Dallas, TX, USA). Protein marker was received from Thermo Fisher Scientific (Waltham, MA, USA) ECL solution for immunoblotting was obtained from Millipore Corporation (Burlington, MA, USA). Ceramide and S1P ELISA kits were received from My BioSource (San Diego, CA, USA). Mebendazole (catalog No. S4610, purity: 99.67 %) powder forms were received from Selleck chemicals LLC (Houston, TX, USA). 

### 5.2. Cell Lines

Cell lines (PANC-1, Capan-1, and MIA Paca-2) were received from a Korean cell bank and were maintained in DMEM medium with 10% FBS (heat inactivated) and 1% PS. Seed the cells on a 6, 12, 96, or 24 well plate according to the experiment and incubated the cells at 37 °C with 5% CO_2_ until confluence reached 80–90%. The treatments were performed after 24 h of cell seeding.

### 5.3. MTT Assay

MTT assay was used to assess PDAC cell viability in response to various chemicals. Cells were seeded in 96 well plates (2500 cells/well), incubated for 24 h at 37 °C with 5% CO_2_, and then treated with the required drug concentration for another 24 h before adding 10 μL of MTT reagent (EZ-CYTOX, DoGenBio Co., Ltd., EZ-3000, South Korea) and incubating for 90 min. The absorbance was taken at 450 nm using the Thermo Scientific Multiskan GO (Waltham, MA, USA). An assay was performed in triplicate.

### 5.4. Colony-Forming Assay

The colony-forming assay was performed on six well plates, in each of which 1000 cells were seeded. After 24 h, the required concentration of drug treatment was performed and incubated for 24 h. Then the older medium was replaced by a fresh medium without drugs, which was incubated for about 14 days, intermittently changing the fresh medium when required. Cells were fixed for 2 min in 4% formaldehyde (Sigma Aldrich, Burlington, MA, USA) and stained for 25 min in 0.5% crystal violet in 25% α -ketoglutaraldehyde, washed to unbind excess stain, dried and photographed. Colonies were counted using the ImageJ software (Version 1.53).

$$\left[\mathrm{Plating}\;\mathrm{efficiency}\;\left(\mathrm{PE}\right)\right]=\frac{\mathrm{Number}\;\mathrm{of}\;\mathrm{colonies}\;\mathrm{in}\;\mathrm{control}}{\mathrm{Number}\;\mathrm{of}\;\mathrm{cell}\;\mathrm{seeded}}$$

$$\left[\mathrm{Surviving}\;\mathrm{Fraction}\right]=\frac{\mathrm{Number}\;\mathrm{of}\;\mathrm{colony}\;\mathrm{counted}\;}{\mathrm{Number}\;\mathrm{of}\;\mathrm{cell}\;\mathrm{seeded}\;\times \mathrm{PE}\;}$$

### 5.5. Transwell Assay for Migration

Prior to cell seeding, all transwell inserts were soaked in FBS-free media for 1 h. In the upper chamber, cells were seeded (5 × 10^4^ cells/well) without FBS 0.3 mL medium, and in the lower chamber, 10% FBS 0.6 mL medium was added. After 4 h of incubation, the respective compounds were treated in the upper chamber with the required concentration and incubated for the next 24 h at 37 °C. Following that, the medium was removed from both chambers, washed with PBS, and cells were stained and fixed with methanol for 20 min before being stained with 0.1% crystal violet in 25% α-ketoglutaraldehyde for 30 min. All inserts were rinsed in PBS (1X) and non-migrated cells were cleared away from the upper transwell membrane with a PBS-soaked cotton bud, and then dried at room temperature. Images were captured using an inverted DMi1 Leica microscope (Wetzlar, Hesse, Germany). From each transwell, five pictures were captured from five different microscopic fields.

### 5.6. Annexin V-FITC Assay

According to the manufacturer’s guidance, annexin V-FITC and propidium iodide (PI) staining were used to identify cell apoptosis. In 12-well plates, MIA Paca-2 (2 × 10^5^ cells/well) were plated for 24 h. Following that, cells were exposed to various compounds, including the necessary concentration of MBZ for 24 h, and annexin V-FITC staining was used to determine if cells had undergone apoptosis. 

### 5.7. ELISA Assay

For the detection of S1P and ceramide level, sandwich ELISA was carried out according to the protocol provided by the ELISA kit (S1P MBS2516132 and ceramide 7254089). Briefly, protein extraction for the ELISA test was prepared according to kit protocol, and absorbance was taken by using a Thermo Scientific Multiskan GO machine at 450 nm.

### 5.8. SK Assay

SK1/2 activity was measured using a 20 μM concentration of each compound using an AdaptaTM screening system (Thermo Fisher Scientific system, Waltham, MA, USA). The SK1 activity assay used 0.04–0.16 ng SK1, 50 μM sphingosine lipid substrate in 32.5 μM HEPES pH 7.5, 0.005% BRIJ 35, 5 μM MgCl_2_, 0.5 mM ethylene glycol-bis (*β*-aminoethyl ether)-*N*, *N*, *N*′, *N*′-tetraacetic acid (EGTA). The SK2 activity assay detected 35–140 ng SK2, 50 μM sphingosine lipid substrate in 32.5 μM HEPES pH 7.5, 0.5 μM EGTA, 1.5 μM MgCl_2_. 

### 5.9. Immunoblotting

The MIA Paca-2 cells were seeded in 6-well plates (1 × 10^6^ cells/well) and treated with different drugs at a 20 µM concentration, including MBZ, followed by another 24 h of incubation. Protein was extracted containing mammalian protein extraction buffer (GE Healthcare Bioscience, Piscataway, NJ, USA) and protease and phosphatase inhibitor (Thermo Scientific, 1861281, USA). Proteins were quantified by BCA (Thermo Scientific, Rockford, USA) and western blotting (SDS-PAGE) was done using 10–30 µg/lane protein sample. Proteins were transferred to a PVDF membrane (Merck Millipore Ltd., Tullagreen, Carrigtwohill, Ireland) and, blocked for 2 h using 3.5% skimmed milk. The blotting membrane was incubated with anti- β-actin, anti-PARP, anti-Bcl-2, anti-Caspase-3, anti-Bax, anti-JAK2, anti-p-JAK2, anti-STAT3, anti-p-STAT-3, anti-E-cadherin, anti-vimentin, anti-FAK, anti-p-FAK, anti-SK2 and anti-SK1 antibodies in the ratio of 1:3000 dilutions. HRP-conjugated anti-mouse, anti-goat, and anti-rabbit IgG secondary antibodies were used in the dilution of 1:5000 and finally proteins were detected by the use of chemiluminescence HRP substrate in Amersham^TM^ imager 680.

### 5.10. JC-10 Assay

JC-10 was used to determine the mitochondrial membrane potential of a cell. This procedure was carried out in accordance with the instructions published by Thermo Fisher Scientific. In summary, 2.5 × 10^5^ cells/well in a 12-well plate were seeded, and then the drugs were administered at the appropriate concentration of 20 µM after 24 h of incubation. A FACS machine with an Arthur image-based interface was used to assess the data after the JC-10 stain had been applied for 30 to 60 min at room temperature.

### 5.11. Phase Contrast Microscopy for Cell Morphology Change 

2 × 10^5^ MIA PaCa-2 cells per well were seeded on 12-well plates and incubated at 37 °C for 24 h with 5% CO_2_ -supplied incubator. After that, cells were treated with 20 µM chemicals (MBZ and RB005) alone or in combination with 5 µM S1P and further incubated for 24 h. Then, the older medium was aspirated out and washed cells with 1X PBS twice and images were taken using an inverted DMi1 Leica microscope (Wetzlar, Hesse, Germany) at 10× objectives.

### 5.12. S1P Preparation and Cell Treatment 

The manufacturer’s instructions were followed to prepare S1P, which was obtained from Echelon Biosciences Inc. (Salt Lake City, USA) under catalogue number S-2000. To summarize, S1P was dissolved in pure methanol, thoroughly mixed by vortexing, and then aliquoted in 1.5 mL tubes and kept at −20 °C. To get the needed concentration on the day of the drug treatment, the S1P was conjugated with fatty acid-free BSA (4 mg/mL) in the desired volume, incubated at 37 °C, and well mixed by a vortex.

### 5.13. Statistical Analysis

Statistical analysis was carried out using the GraphPad Prism 8.4 software (GraphPad Software, San Diego, CA, USA). To compare the means of two or more groups one-way analysis of variance (ANOVA) was used and compared the data using a multiple comparison test. Mean ± standard deviations (SD) were used to indicate bars and error. Statistical significance of data was measured by *p value < 0.05, ***p < 0.01. Each experiment was carried out three times independently.

## Data Availability

Not applicable.
